# Spin transport in non-Hermitian quantum systems

**DOI:** 10.1038/s41598-023-38293-5

**Published:** 2023-07-10

**Authors:** Leonardo S. Lima

**Affiliations:** Department of Physics, Federal Center for Technological Education of Minas Gerais, 30510-000 Belo Horizonte, MG Brazil

**Keywords:** Magnetic properties and materials, Phase transitions and critical phenomena, Spintronics

## Abstract

Transport in non-Hermitian quantum systems is studied. The goal is a better understanding of transport in non-Hermitian systems like the Lieb lattice due to its flat bands and the integrability of the Ising chain which allows transport in that model to be computed analytically. This is a very special feature that is not present in a generic non-Hermitian system. We obtain the behaviour of the spin conductivity as a function of the non-Hermitian parameters of each system with aim to verify the influence of variation them on conductivity. For all models analyzed: Ising model as well as noninteracting fermion models, we obtain a little influence of the non-Hermitian parameters on conductivity and thus, a small effect over transport coefficients. Furthermore, we obtain an influence of opening of the gap in the spectrum in these models on longitudinal conductivity as well.

## Introduction

Non-Hermitian quantum mechanics is an extension of the standard quantum mechanics^1–5^. It might provide a description of dissipative quantum systems although it is not the universal description for them. Despite the fact that the operators of physical observable are required to be Hermitian, the Hermiticity can be relaxed in a $\textbf{n}$-pseudo Hermiticity^6–9^ or time-reversal symmetry (PT) in the non-Hermitian quantum mechanics, where $\textbf{n}$ is a linear (or anti-linear) Hermitian operator and anti-Hermitian operator, and *P*, *T* stand for the parity and time-reversal operators respectively^10–13^. In the early of 40 decade, non-Hermitian Hamiltonian operators were introduced by Dirac^14^ and Pauli^15^ and an $\textbf{n}$-dependent indefinite metric in the Hilbert space in order to deal with some divergence problems related to unitarity of time evolution (conservation of probability). In the late 60s, non-Hermitian Hamiltonians were applied to quantum electrodynamics for keeping unitarity of the *S*-matrix^16^. Since then many other authors revealed that a non-Hermitian Hamiltonian could have real eigenvalues under specific conditions.

Recently, a quantity of inspiring studies in non-Hermitian physics has risen rapidly in condensed matter physics. Non-Hermitian models and exotic quantum many-body effects as non-Hermitian extensions of the Kondo effect, fermionic superfluidity have been reported in non-Hermitian quantum spin systems^17–21^. Furthermore, non-Hermitian topological phases have gained a large interest due to their unique properties. One of the most intriguing is the skin effect or the localization of a macroscopic fraction of bulk eigenstates at a boundary, that underlies the breakdown of the bulk-edge correspondence and that has been uncovered in photonic systems and materials with non-Hermitian interactions^22–26^. There are also some other works discussing exception points in many-body systems^27–39^. All these studies unveil interesting and important effects of non-Hermiticity in interacting systems. The research in non-Hermitian physics has been greatly developed in the non-Hermitian phase transition point in non-Hermitian systems which plays a important role in the its dynamics^2^.

The goal of the present paper is to study transport in non-Hermitian models like non-interacting fermion model and the one-dimensional Ising model. The symmetry-protected non-Hermitian transport in the lattice models is systematically discussed in the literature^40^, which shows the fundamental guiding principles of non-Hermitian quantum transport and light propagation. Notably, we find a large influence on single-particle energy spectrum, which can be obtained by the same procedure and that will have the same value when the system approaches of the thermodynamic limit, generating an effect on DC and AC conductivities. This paper is organized as follows: In “Non-Hermitian quantum spin systems” section, we discuss about the models. In “Transport” section, the longitudinal spin conductivity is studied in the framework of the linear response theory. In the last section, “Summary”, we present our conclusions and final remarks.

## Non-Hermitian quantum spin systems

### Two-dimensional non-Hermitian Lieb lattice

An example of a non-Hermitian (non-self-adjoint) quantum system is the model of non-interacting 2D fermions in the Lieb lattice

1 $$\begin{aligned} \mathcal {H} & = \sum _{m,n}\{[\nu ( \alpha ^{\dag }_{mn}\beta _{mn}+ \beta ^{\dag }_{mn}\gamma _{mn} + \alpha ^{\dag }_{mn}\beta _{m+1, n}+ \beta ^{\dag }_{mn}\gamma _{m,n+1})\nonumber \\ & \quad +i\mu \gamma _{mn}^{\dag }\alpha _{mn}+\hbox {H.c.}] +i\varepsilon \left( \alpha ^{\dag }_{mn}\alpha _{mn}-\gamma ^{\dag }_{mn}\gamma _{mn}\right) \}, \end{aligned}$$

which is well adequate for the ultracold atomic gas in optical lattices, photonic crystals and coupled resonators^41^. In this case, the Hamiltonian of the system in the $\textbf{k}$ space is written as

2 $$\begin{aligned} {} \mathcal {H}=\sum _{\textbf{k}}\mathcal {H}({\textbf{k}})=\sum _{\textbf{k}}\Phi ^{\dag }_{\textbf{k}}\Theta ({\textbf{k}})\Phi _{\textbf{k}}, \end{aligned}$$

where the Hamiltonians in the momentum subspaces commute with each other, $\left[ \mathcal {H}({\textbf{k}}),\mathcal {H}({\mathbf {k'}})\right] =0$. $\nu ,\mu ,\varepsilon \in \mathbb {R}$. Moreover, $\Phi _{\textbf{k}}=\left( \alpha _{\textbf{k}},\beta _{\textbf{k}},\gamma _{\textbf{k}}\right) ^{T}$ and

3 $$\begin{aligned} \Theta ({\textbf{k}})=\left( \begin{array}{ccc} i\varepsilon &{} \nu (e^{ik_x}+1) &{} -i\mu \\ \nu (e^{-ik_x}+1) &{} 0 &{} \nu (e^{ik_y}+1) \\ i\mu &{} \nu (e^{-ik_y}+1) &{} -i\varepsilon \\ \end{array} \right) . \end{aligned}$$

**Figure 1:**
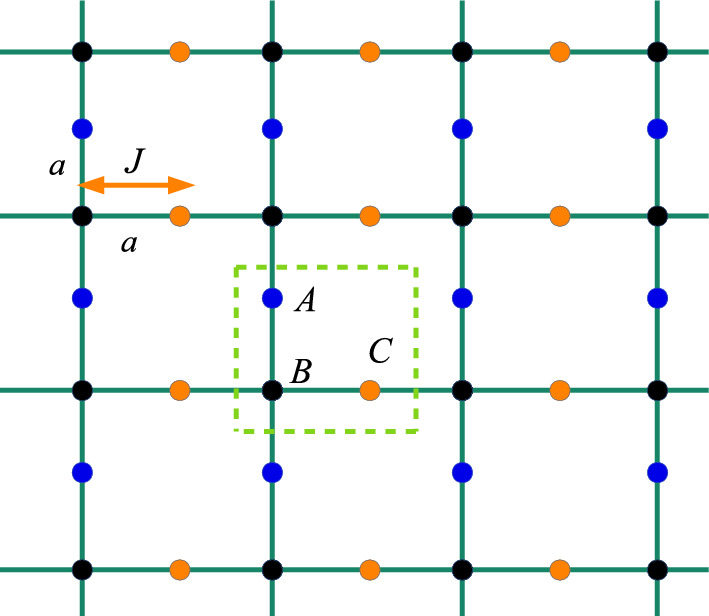
Representation of the Lieb lattice. Each unit cell presents three different sites. The lattice parameter is $a=1$: the vectors $\textbf{a}_1=(a,0)$ and $\textbf{a}_2=(0,a)$ connects the sites *BA* and *BC*, respectively.

A representation of the non-Hermitian model on Lieb lattice is made in Fig. 1, where each unit cell of the lattice presents three different sites. The lattice parameter (distance between the sites *AA*, *BB*) is given by the vectors ($a=1$): $\textbf{a}_1=(a,0)$ and $\textbf{a}_2=(0,a)$, that connects the sites *BA* and *BC*, respectively. The energy bands of $\Pi (\textbf{k})$ are obtained by solving of the cubic polynomial $\det \left( \Theta _{\textbf{k}}-\Pi _{\textbf{k}}\textbf{I}\right) =0$, where $\textbf{I}$ is the identity matrix^41^ and

4 $$\begin{aligned} {}{} & {} \Pi (\textbf{k})^{3}+\left[ \varepsilon ^2-|\nu (e^{ik_x}+1)|^2-|\nu (e^{ik_y}+1)|^2-\mu ^2\right] \Pi (\textbf{k})\nonumber \\{} & {} \quad +i\varepsilon \left[ |\nu (e^{ik_y}+1)|^2-|\nu (e^{ik_x}+1)|^2\right] \nonumber \\{} & {} \quad +2|\nu (e^{ik_x}+1)||\nu (e^{ik_y}+1)|\mu \sin \left( \frac{k_x+k_y}{2}\right) =0. \end{aligned}$$

The cubic algebraic equation can be solved exactly by radicals whose solution is given by

5 $$\begin{aligned} {} \Pi (\textbf{k})=\root 3 \of {-\frac{\mathfrak {a}(\textbf{k})}{2}+\Xi (\textbf{k})} +\root 3 \of {-\frac{\mathfrak {a}(\textbf{k})}{2}-\Xi (\textbf{k})}, \end{aligned}$$

where

6 $$\begin{aligned} \mathfrak {a}(\textbf{k}) & =i\varepsilon \left[ |\nu (e^{ik_y}+1)|^2-|\nu (e^{ik_x}+1)|^2\right] \nonumber \\ & \quad +2|(e^{ik_x}+1)||(e^{ik_y}+1)|\nu ^2\mu \sin \left( \frac{k_x+k_y}{2}\right) , \\ \mathfrak {b}(\textbf{k}) & =\varepsilon ^2-|\nu (e^{ik_x}+1)|^2-|\nu (e^{ik_y}+1)|^2-\mu ^2 \end{aligned}$$

and the discriminant is given by

7 $$\begin{aligned} \Xi (\textbf{k})=\sqrt{\left( \frac{\mathfrak {a}(\textbf{k})}{2}\right) ^2+\left( \frac{\mathfrak {b}(\textbf{k})}{3}\right) ^3}. \end{aligned}$$

We obtain the complex roots of $\Xi (\textbf{k})$ given by

8 $$\begin{aligned} \Xi (\textbf{k})& =\sqrt{E^2+F^2}\left[ \cos \left( \frac{\theta _2}{2}+n\pi \right) +i\sin \left( \frac{\theta _2}{2}+n\pi \right) \right] \hbox {either}\nonumber \\ \Xi _{1,2}(\textbf{k}) & =\pm \sqrt{E^2+F^2}\left[ \cos \left( \frac{\theta _2}{2}\right) +i\sin \left( \frac{\theta _2}{2}\right) \right] , \end{aligned}$$

where $$n\in \mathbb {Z}$$ and

9 $$\begin{aligned} \theta _2 & =\cos ^{-1}\left( \frac{E(\textbf{k})}{\sqrt{E(\textbf{k})^2+F(\textbf{k})^2}}\right) ,\tan \theta _2=F(\textbf{k})/E(\textbf{k}),\nonumber \\ E(\textbf{k}) & =\frac{1}{4}\left[ A(\textbf{k})^2+B(\textbf{k})^2\right] +\frac{1}{27}\left[ C(\textbf{k})^3-C(\textbf{k})D(\textbf{k})^2\right] ,\nonumber \\ F(\textbf{k}) &=-\frac{1}{27}\left[ 3C(\textbf{k})^2D(\textbf{k})+D(\textbf{k})^3\right] ,\nonumber \\ A(\textbf{k}) & =\varepsilon \nu ^2\left( \sin 2k_x-\sin 2k_y+2\sin k_x-2\sin k_y\right) \nonumber \\ & \quad +2\mu \nu ^2\sin \left( \frac{k_x+k_y}{2}\right) \left[ \cos (k_x+k_y)+\cos k_x+\cos k_y+1\right] ,\nonumber \\ B(\textbf{k}) &=\varepsilon \nu ^2\left( \cos 2k_y-\cos 2k_x+2\cos k_y-2\cos k_x\right) \nonumber \\ & \quad +2\mu \nu ^2\sin \left( \frac{k_x+k_y}{2}\right) \left[ \sin (k_x+k_y)+\sin k_x+\sin k_y+1\right] ,\nonumber \\ C(\textbf{k}) &=\varepsilon ^2-\nu ^2\left( \cos 2k_x+\cos 2k_y+2\cos k_x+2\cos k_y+2\right) -\mu ^2,\nonumber \\ D(\textbf{k}) & =\nu ^2\left( \sin 2k_x+\sin 2k_y+2\sin k_x+2\sin k_y\right) . \end{aligned}$$

Hence, the energy bands are given by

10 $$\begin{aligned} {} \Pi (\textbf{k})=2\root 3 \of {\mathfrak {c}(\textbf{k})^2+\mathfrak {d}(\textbf{k})^2}\cos \left( \frac{\theta _1+2n\pi }{3}\right) , \end{aligned}$$

or

11 $$\begin{aligned} \Pi _1(\textbf{k}) & =2\root 3 \of {\mathfrak {c}(\textbf{k})^2+\mathfrak {d}(\textbf{k})^2}\cos \left( \frac{\theta _1}{3}\right) ,\nonumber \\ \Pi _2(\textbf{k}) & =2\root 3 \of {\mathfrak {c}(\textbf{k})^2+\mathfrak {d}(\textbf{k})^2}\cos \left( \frac{\theta _1+2\pi }{3}\right) ,\nonumber \\ \Pi _3(\textbf{k}) & =2\root 3 \of {\mathfrak {c}(\textbf{k})^2+\mathfrak {d}(\textbf{k})^2}\cos \left( \frac{\theta _1+4\pi }{3}\right) , \end{aligned}$$

where $$-\pi<\frac{\theta _1+2n\pi }{3}<\pi$$ and

12 $$\begin{aligned} \theta _1 &=\cos ^{-1}\left( \frac{\mathfrak {c}(\textbf{k})}{\sqrt{\mathfrak {c}(\textbf{k})^2+\mathfrak {d}(\textbf{k})^2}}\right) , \tan \theta _1=\mathfrak {d}(\textbf{k})/\mathfrak {c}(\textbf{k}),\nonumber \\ \mathfrak {c}(\textbf{k}) &=\sqrt{E^2+F^2}\cos \left( \frac{\theta _2}{2}\right) -\frac{A(\textbf{k})}{2}\nonumber \\ \mathfrak {d}(\textbf{k}) &=\sqrt{E^2+F^2}\sin \left( \frac{\theta _2}{2}\right) -\frac{B(\textbf{k})}{2}. \end{aligned}$$

**Figure 2:**
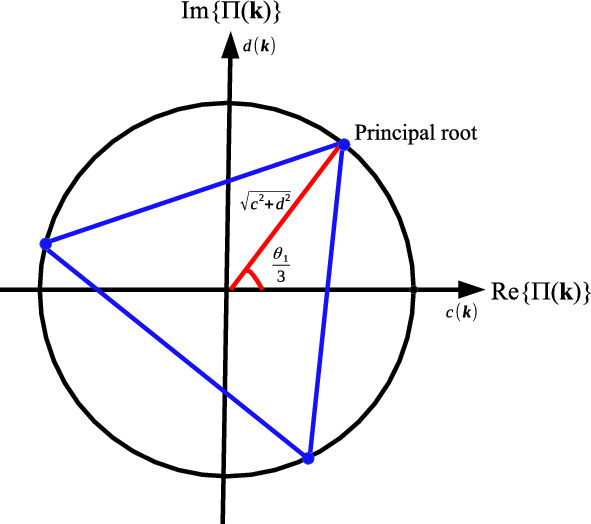
Distribution of the energy bands Eq. (11) on complex plane.

The representation of the energy bands $\Pi (\textbf{k})$, on complex plane is displayed in Fig. 2. The behavior of $\Xi (\textbf{k})$ induced by the coupling parameters of the non-Hermitian model will generate a large influence on continuum and DC conductivities.

### Non-Hermitian Ising model

The model is given by^42^

13 $$\begin{aligned} {} \mathcal {H}=\sum _{j=1}^{N}\sigma _{j}^z\sigma _{j+1}^z+\lambda (\sigma _{j}^x+i\delta \sigma _j^y). \end{aligned}$$

For periodic boundary condition $\sigma _j^{x}=\sigma _{j+L}^{x}$, $\sigma _j^{y}=\sigma _{j+L}^{y}$ and $\sigma _j^{z}=\sigma _{j+L}^{z}$, the Hamiltonian can be transformed in the form

14 $$\begin{aligned} {} \tilde{\mathcal {H}}=\sum _{j=1}^{N}T_{j}^zT_{j+1}^z+\lambda T_{j}^x(1-\delta ^2)^{1/2}, \end{aligned}$$

which is the standard Ising model with a transverse field $\lambda \sqrt{1-\delta ^2}$ that holds if and only if $|\delta |\ne 1$. Performing the Jordan-Wigner transformation

15 $$\begin{aligned}{} & {} T_j^x =\frac{1}{2}-\bar{g}_jg_j, \nonumber \\{} & {} T_j^y =\frac{i}{2}\sum _{j<l}(1-2\bar{g}_jg_j)(\bar{g}_j-g_j), \nonumber \\{} & {} T_j^{z}=-\frac{1}{2}\sum _{j<l}(1-2\bar{g}_jg_j)(\bar{g}_j+g_j) \end{aligned}$$

to replace the quasispin operators by the new non-Hermitian operators $g_j$ and $\bar{g}_j$, where $\bar{g}_j=\mathfrak {A}_jc_j^{\dag }\mathfrak {A}_j^{-1}$, $g_j=\mathfrak {A}_jc_j\mathfrak {A}_j^{-1}$, $c_j^{\dag }$, $c_j$ are the creation and annihilation operators of spinless fermions. The new operators satisfy the fermionic anticommutation relation $\{\bar{g}_j,g_j'\}=\delta _{j,j'}$. We set $\{|\psi _n\rangle \}$ as the eigenstates of the operator $\sum _i\sigma ^z_j$ that represents all possible spin configurations along the $+z$ direction. To proceed, one introduces a similarity transformation $\mathfrak {A}=\prod _j\mathfrak {A}_j$, where $\mathfrak {A}_j=e^{-i\theta \sigma ^z_j}$, represents a counterclockwise spin rotation in the $\sigma _x-\sigma _y$ plane around the $\sigma _z$ axis by an angle $\theta$: $\theta =\tan ^{-1}(i\gamma )$, which is a complex number that depends on the strength of the complex field. Under the biorthogonal basis of $\{\mathfrak {A}_j^{-1}|\psi _n\rangle \}$ and $\{\mathfrak {A}_j^{\dag }|\psi _n\rangle \}$, the matrix form of $\tilde{\mathcal {H}}$ is Hermitian for $|\delta |<1$. The parity of the number of fermions is a conservative quantity such that the Hamiltonian can be expressed as $\tilde{\mathcal {H}}=\tilde{\mathcal {H}}_{+}\textbf{I}=\tilde{\mathcal {H}}_{-}\textbf{I}$, where $\tilde{\mathcal {H}}_{+}=\tilde{\mathcal {H}}_{-}=-2(\bar{g}_{N}\bar{g}_1+\bar{g}_{N}g_1+\bar{g}_1g_{N}+g_1g_{N})$ and the Hamiltonian is rewritten as

16 $$\begin{aligned} \bar{\mathcal {H}} & =\frac{1}{4}\sum _{j=1}^{N}2[\lambda \sqrt{1-\delta ^2}(1-2\bar{g}_jg_j)\nonumber \\ & \quad +(\bar{g}_j\bar{g}_{j+1}+\bar{g}_jg_{j+1}+\bar{g}_{j+1}g_{j}+g_{j+1}g_{j})]. \end{aligned}$$

**Figure 3:**
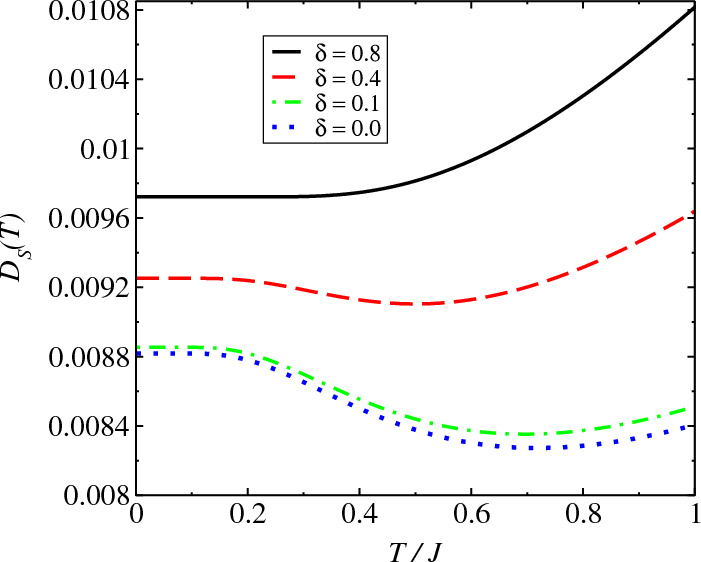
Behavior of the Drude’s weight $D_S(T)$ as a function of *T* for the Ising model, Eq. (13). For $\delta =0$ we have the Hermitian model and for $\delta \ne 0$, the model is non-Hermitian. We obtain a finite Drude weight at $T=0$ indicating an ideal spin conductor at $T=0$. We consider $\lambda =1.0$ in the calculations.

Taking the discrete Fourier transform

17 $$\begin{aligned} g_j=\frac{1}{\sqrt{N}}\sum _{\textbf{k}}g_{\textbf{k}}e^{i{\textbf{k}\cdot \textbf{r}_j}}, \bar{g}_j=\frac{1}{\sqrt{N}}\sum _{\textbf{k}}g_{\textbf{k}}e^{-i{\textbf{k}\cdot \textbf{r}_j}}, \end{aligned}$$

where $|\textbf{k}|=k=2\pi (n+1/2)/N$, $n=0,1,2,...,N-1$, the Hamiltonian can be written as

18 $$\begin{aligned} \bar{\mathcal {H}}_{+}=\bar{\mathcal {H}}_{-}=\sum _{0<k<\pi }\bar{\chi }_{\textbf{k}}\bar{\mathcal {H}}_{+}(\textbf{k})\chi _{\textbf{k}}, \end{aligned}$$

with $\bar{\chi }_{\textbf{k}}=(\bar{g}_{\textbf{k}},g_{-\textbf{k}})$, $\chi _{\textbf{k}}=(g_{\textbf{k}},\bar{g}_{-\textbf{k}})^{T}$. Making the non-Hermitian Bogoliubov transformation

19 $$\begin{aligned} \bar{\psi }_{\textbf{k}}= & {} \cos \frac{\varrho _{\textbf{k}}}{2}\bar{g}_{\textbf{k}}+i\sin \frac{\varrho _{\textbf{k}}}{2}g_{-\textbf{k}}, \end{aligned}$$

20 $$\begin{aligned} \psi _{\textbf{k}}= & {} \cos \frac{\varrho _{\textbf{k}}}{2}g_{\textbf{k}}-i\sin \frac{\varrho _{\textbf{k}}}{2}\bar{g}_{-\textbf{k}}, \end{aligned}$$

where $[\bar{\psi }_{\textbf{k}},\psi _{\mathbf {k'}}]=\delta _{\textbf{k},\textbf{k}'}$ and $\varrho _{\textbf{k}}=\tan ^{-1}[\sin (\textbf{k})/(2\lambda \sqrt{1-\delta ^2}-\cos (\textbf{k}))]$. The Hamiltonian is recast in the diagonal form with the dispersion relation of quasi-particles given by

21 $$\begin{aligned} \omega _{\textbf{k}}=\sqrt{4\lambda ^2(1-\delta ^2)-4\lambda \cos (\textbf{k})(1-\delta ^2)^{1/2}+1}. \end{aligned}$$

If $|\delta |< 1$, the single-particle energy is real and $|\delta |> 1$, the system presents a complex single-particle spectrum regardless of $\textbf{k}$.

## Transport

**Figure 4:**
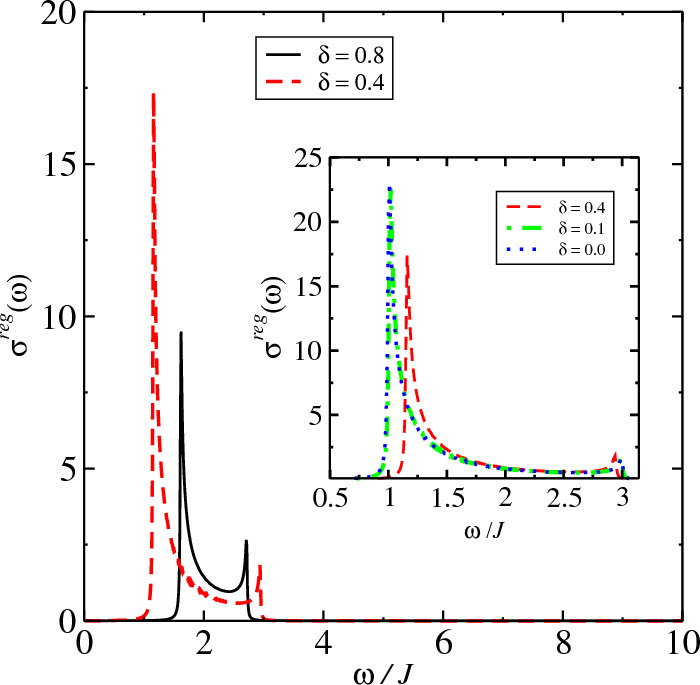
$\sigma ^{reg}(\omega )$ at $T=0.01J$ for different values of non-Hermitian coupling $\delta$ for the Ising model, Eq. (13). For $\delta =0$ we have the Hermitian model and $\delta \ne 0$, the model is non-Hermitian. We find that conductivity tends to zero at DC limit. We make $\lambda =1.0$ in the calculations.

In the linear response theory for Hermitian systems, the response of the system to the frequency-dependent gradient of the external magnetic field $\textbf{h}$ generates a spin current given by $\mathcal {J}=\sigma \nabla \textbf{h}$, where the response linear to the external field in *x* direction is

22 $$\begin{aligned} \langle \mathcal {J}_x(l,t)\rangle =\sum _{j}\int _{-\infty }^{\infty }dt'\chi _{jS}(l,j,t-t')h^z(j,t'), \end{aligned}$$

being the response function defined as

23 $$\begin{aligned} \chi _{jS}(l,j,t-t')\equiv i\Theta (t-t')\langle 0|[\mathcal {J}_x(l,t),S^z(j,t')]|0\rangle , \end{aligned}$$

where $\Theta$ is the Heaviside step function. On the other hand, the non-Hermitian response function is given by^43^

24 $$\begin{aligned} \chi _{jS}^{NH}(l,j,t-t') & =-\frac{1}{\hbar }\Theta (t-t')\bigg [\langle 0|\{\mathcal {J}_x(l,t),S^z(j,t')\}|0\rangle \nonumber \\ & \quad -2\langle 0|\mathcal {J}_x(l,t)|0\rangle \langle 0|S^z(j,t')|0\rangle \bigg ], \end{aligned}$$

where $\{\cdot \cdot \cdot \}$ is the unequal-time anti-commutator to establish the link between the response function and the correlation function. We have the non-Hermitian dynamic susceptibility as the Fourier transform

25 $$\begin{aligned} \chi _{jS}^{NH}(\tau ,\omega )=\int _{-2\tau }^{2\tau }d\Delta t\chi _{jS}^{NH}\left( \tau +\frac{\Delta t}{2},\tau -\frac{\Delta t}{2}\right) e^{i\omega \Delta t}, \end{aligned}$$

where $\tau =it$. $\chi _{jS}$ is the non-Hermitian response function

26 $$\begin{aligned} \chi _{jS}^{NH}(t,t')=\frac{i}{\hbar } \Theta (t-t')\langle 0|\{\mathcal {J}_x(t),S^z(t')\}|0\rangle . \end{aligned}$$

The wave-vector-dependent susceptibility is given by

27 $$\begin{aligned} \chi _{jS}^{NH}(\textbf{k},\omega )\equiv \frac{i}{N}\int _{0}^{\infty }dte^{i(\omega +i0^{+})t} \langle 0|\{\mathcal {J}_x(\textbf{k},t),S^z(-\textbf{k},t')\}|0\rangle . \end{aligned}$$

From continuity for the spin current:

$\dot{S}^z(\textbf{k},t)+i\textbf{k}\cdot \mathcal {J}_x(\textbf{k},t)=0$, $\chi _{jS}^{NH}$ can be transformed as follows:

28 $$\begin{aligned} \chi _{jS}^{NH}(\textbf{k},\omega ) & =\frac{i}{N}\frac{1}{i(\omega +i0^{+})}\bigg [ik_x\int _{0}^{\infty }dte^{i(\omega +i0^{+})t}\nonumber \\ & \quad \times \langle 0|\{\mathcal {J}_x(\textbf{k},t),\mathcal {J}_x(-\textbf{k},0)\}|0\rangle \nonumber \\ & \quad -\langle 0|\{\mathcal {J}_x(\textbf{k},0),S^z(-\textbf{k},0)\}|0\rangle \bigg ]. \end{aligned}$$

Using the representation of the spin current operator in terms of spin operators

29 $$\begin{aligned} \mathcal {J}_x(j)=\frac{iJ}{2}\sum _x(S_{j}^{+}S_{j+x}^{-}-S_{j}^{-}S_{j+x}^{+}), \end{aligned}$$

where $j+x$ is the nearest-neighbor site of the site *j* in the positive *x* direction, one can transform the second term as

30 $$\begin{aligned} \langle 0|\{\mathcal {J}_x(\textbf{k},0),S^z(-\textbf{k},0)\}|0\rangle & = \frac{i}{2}\sum _{l,x}\mathcal {J}_{l,l+x}\left( e^{ik_x}-1\right) \nonumber \\ & \quad \times \langle S_{j}^{+}S_{j+x}^{-}+S_{j}^{-}S_{j+x}^{+}\rangle . \end{aligned}$$

In the long-wavelength $k_x\rightarrow 0$ limit the susceptibility $\chi _{jS}^{NH}(\textbf{k},\omega )$ is thus proportional to $ik_x$ and we can write

31 $$\begin{aligned} \langle \mathcal {J}_{x}(\textbf{k},\omega )\rangle =-\frac{\langle -K_x\rangle -\mathfrak {G}(\textbf{k},\omega )}{i(\omega +i0^{+})}ik_xh^z(\textbf{k},\omega ), \end{aligned}$$

where $\langle -K_x\rangle$ is the kinetic energy, being given by

32 $$\begin{aligned} \langle \textrm{K}_x \rangle =-\frac{J}{N}\sum _{j}\left( S_j^{+}S_{j+x}^{-}+S_j^{-}S_{j+x}^{+}\right) , \end{aligned}$$

and $\mathfrak {G}$ is the Green’s function defined in $T=0$ by^44^

33 $$\begin{aligned} \mathfrak {G}({\textbf{k}},\omega )=\frac{i}{ N}\int _{0}^{\infty }dte^{i\omega t}\langle 0| [\mathcal {J}_x({\textbf{k}},t),\mathcal {J}_x(-{\textbf{k}},0)]|0\rangle , \end{aligned}$$

being ${\textbf{T}}$, the time ordering operator.

The regular part of the conductivity $\sigma$ (continuum conductivity) in the context of Hermitian quantum mechanics is given by^44–49^: $\hbox {Re}\left[ \sigma (\omega )\right] =D_S(T)\delta (\omega )+\sigma ^{reg}(\omega )$, where

34 $$\begin{aligned} \langle \mathcal {J}_{\alpha }(\textbf{k},\omega )\rangle & =\sum _{\beta }\sigma _{\alpha \beta }(\textbf{k},\omega )ik_{\alpha } h_{\beta }(\textbf{k},\omega ),\nonumber \\ \sigma _{\alpha \beta }(\textbf{k},\omega ) & =\hbox {Re}[\sigma _{\alpha \beta }(\textbf{k},\omega )]+i\hbox {Im}[\sigma _{\alpha \beta }(\textbf{k},\omega )]\nonumber \\ \sigma ^{reg}(\omega ) & =\frac{\hbox {Im} \{\mathfrak {G}({\textbf{k}}=0,\omega )\}}{\omega } \end{aligned}$$

and $\alpha ,\beta =x,y,z$. $D_S(T)$ is the spin Drude’s weight, being given by

35 $$\begin{aligned} D_S(T)\approx -\frac{1}{N}\sum _{\textbf{k}}\frac{\cos (k_{x})}{\omega _{\textbf{k}}}[1+n(\omega _{\textbf{k}})], \end{aligned}$$

where $n(\omega _{\textbf{k}})=1/(e^{\beta \omega _{\textbf{k}}}\pm 1)$ is the occupation number of bosons and fermions and $\beta =1/T$.

The behavior of Drude’s weight $D_S(T)$ as a function of *T* is displayed in Fig. 3 for the Ising model Eq. (13). The effective *T* that best relates the susceptibilities via fluctuation dissipation relation for a fixed waiting time $t_w$ is given as^43^

36 $$\begin{aligned} {} T= & {} \arg \min _{\Theta }\int d\omega \left[ -\chi '^{NH}(t_w,\omega )\tanh \left( \frac{\hbar \omega }{2k_B\Theta }\right) -\chi ''(t_w,\omega )\right] ^2, \end{aligned}$$

where $\chi ^{NH}=\chi '^{NH}+i\chi ''^{NH}$, $\chi =\chi '+i\chi ''$. For $\delta =0$ we have the Hermitian model and $\delta \ne 0$ the model is non-Hermitian. We obtain a small difference in the behavior of the curves for the two models (Hermitian and non-Hermitian) due to transformation of the non-Hermitian Hamiltonian in Hermitian, Eq. (14). Moreover, for *T* non-zero, $D_S(T)$ rises with *T* however, this description is only qualitative due to approach used.

The continuum part of the spin conductivity $\sigma ^{reg}(\omega )$, is defined in terms of the Green’s function $\mathfrak {G}(\omega )$.

**Figure 5:**
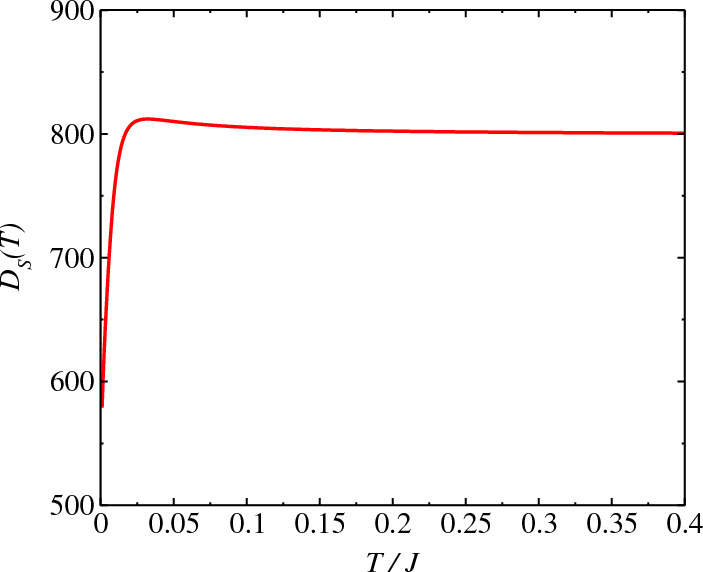
Drude’s weight $D_S(T)$ as a function of *T* for small values of non-Hermitian couplings $\varepsilon ,\mu ,\eta \approx 0.1$, for the 2D non-Hermitian Lieb lattice model Eq. (1). The Drude’s weight is finite for all *T*/*J* indicating thus, an ideal conductor for all *T* values.

We obtain the spin current operator in terms of the operators $\psi ^{\dag }$ and $\psi$ given by

37 $$\begin{aligned} \mathcal {J}_x(t)= -\sum _{\textbf{k}}\frac{\sin (k_{x})}{\omega _{\textbf{k}}}\psi _{\textbf{k}}^{\dag }\mathcal {N}_{\textbf{k}}(t)\psi _{\textbf{k}}.\nonumber \\ \end{aligned}$$

The spin current response function $\mathfrak {G}({\textbf{k}},\omega )$ at non-zero *T* is given by^44^

38 $$\begin{aligned} \mathfrak {G}({\textbf{k}},\omega )=\frac{i}{ N}\int _{0}^{\infty }dte^{i\omega t}\langle 0| [\mathcal {J}_x({\textbf{k}},t),\mathcal {J}_x(-{\textbf{k}},0)]|0\rangle , \end{aligned}$$

where $\mathfrak {G}({\textbf{k}}=0,\omega \rightarrow 0)$ is the susceptibility or retarded Green’s function^44^. The retarded Green’s function or dynamical correlation function is obtained after performing an analytical calculation, where we obtain the result

39 $$\begin{aligned} \mathfrak {G}({\textbf{k}},\omega )=\sum _{\textbf{k},\textbf{k}'}\frac{\sin (k_{x}')}{\omega _{\textbf{k}'}}\frac{\sin (k_{x})}{\omega _{\textbf{k}}}\mathcal {N}_{\textbf{k}}(\omega ),\nonumber \\ \end{aligned}$$

being

40 $$\begin{aligned} \mathcal {N}_{\textbf{k}}(\omega )=\frac{1}{\pi ^2}\int _{0}^{2\pi }d\omega _1\mathfrak {G}_0(\textbf{k},\omega _1)\tilde{\mathfrak {G}}_0(\textbf{k},\omega -\omega _1),\nonumber \\ \end{aligned}$$

and $\mathfrak {G}_0$, $\tilde{\mathfrak {G}}_0$ are the bare propagator.

41 $$\begin{aligned} \mathfrak {G}_0(\textbf{k},\omega )= \frac{1}{\omega -\omega _{\textbf{k}}+i0^{+}},\hspace{0.5cm}\tilde{\mathfrak {G}}_0(\textbf{k},\omega )= \frac{-1}{\omega +\omega _{\textbf{k}}-i0^{+}}.\nonumber \\ \end{aligned}$$

$\mathcal {N}_{\textbf{k}}(\omega )$ is the Fourier transform of $\mathcal {N}_{\textbf{k}}(t)$, which is the dynamical correlation function

42 $$\begin{aligned}{} & {} \mathcal {N}_{\textbf{k}}(t)-i\langle 0|\bigg [\psi _{\textbf{k}}(t)\mathbf{{\sigma }_{\alpha \beta }}\psi ^{\dag }_{\textbf{k}}(t)\psi _{\textbf{k}}(0)\mathbf{{\sigma }_{\alpha \beta }}\psi ^{\dag }_{\textbf{k}}(0)\nonumber \\{} & {} \quad +\psi _{\textbf{k}}(t)\mathbf{{\sigma }_{\alpha \beta }}\psi ^{\dag }_{\textbf{k}}(t)\psi _{\textbf{k}}(0)\mathbf{{\sigma }_{\alpha \beta }}\psi ^{\dag }_{\textbf{k}}(0)+\psi _{\textbf{k}}(t)\mathbf{{\sigma }_{\alpha \beta }}\psi ^{\dag }_{\textbf{k}}(t)\psi _{\textbf{k}}(0)\mathbf{{\sigma }_{\alpha \beta }}\psi ^{\dag }_{\textbf{k}}(0)\nonumber \\{} & {} \quad +\psi _{\textbf{k}}(t)\mathbf{{\sigma }_{\alpha \beta }}\psi ^{\dag }_{\textbf{k}}(t)\psi _{\textbf{k}}(0)\mathbf{{\sigma }_{\alpha \beta }}\psi ^{\dag }_{\textbf{k}}(0)\bigg ]|0\rangle .\nonumber \\ \end{aligned}$$

Consequently, we obtain the regular part of the longitudinal spin conductivity $\sigma ^{reg}(\omega )$ as being given by

43 $$\begin{aligned} \sigma ^{reg}(\omega )=\sum _{\textbf{k}}\sin ^2(k_{x}) \frac{\left[ 1+2n(\omega _{\textbf{k}})\right] }{\omega _{\textbf{k}}^3}\delta (\omega -\omega _{\textbf{k}}).\nonumber \\ \end{aligned}$$

In all cases analyzed, the influence of dispersionless flat modes on longitudinal spin conductivity is only to give rise to a Dirac’s delta-like peak at frequency $\omega =\omega _{\textbf{k}}$, where $\omega _{\textbf{k}}$ is a plane mode in each case. Furthermore, the presence of large peaks in the AC spin conductivity and a finite Drude’s weight $D_S(T)$, indicate a supercurrent behavior for the system although, for one has a superconductor behavior is necessary that the system exhibits the Meissner effect as well^50^.

**Figure 6:**
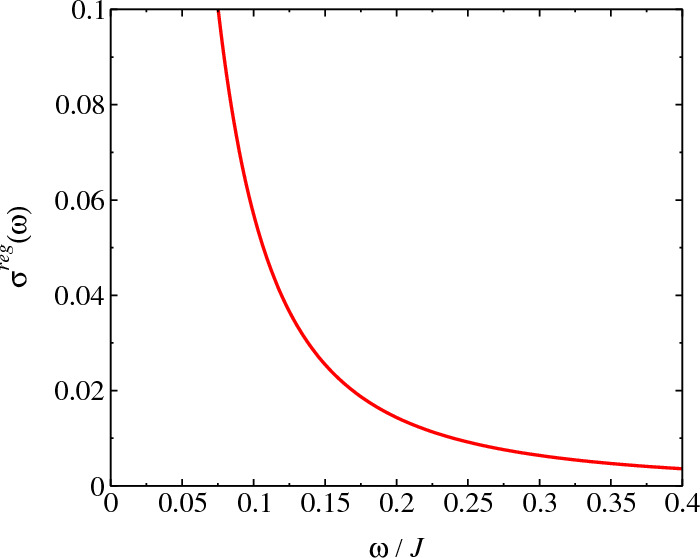
$\sigma ^{reg}(\omega )$ at $T=0.0$ for small values of non-Hermitian couplings $\varepsilon ,\mu ,\eta =0.1$ for the 2D non-Hermitian Lieb lattice model Eq. (1). We obtain the conductivity tending to the infinity at DC limit, indicating thus an ideal transport in this limit.

In Fig. 4, we present the behavior of $\sigma ^{reg}(\omega )$ for different values of non-Hermitian coupling $\delta$. We obtain the AC conductivity tending to zero at $\omega \rightarrow 0$ however, as we have $\sigma (0)=D_S\delta (\omega )$ and since that we obtain a $D_S$ finite, we must have a divergence for the DC current. However, the scattering among particles must introduce a spreading in the conductivity where in a real system the conductivity must to stay finite. The large peaks obtained for the conductivity are due to the behavior of the dispersion relation at range $(1.0<\omega /J<3.0)$, generating so, resonance effects on conductivity. In Figs. 5 and 6, we analyze the conductivity for the non-Hermitian model Eq. (2). In this case, one obtains a divergence in the continuum conductivity at DC limit, $\omega \rightarrow 0$. The behavior obtained for the AC conductivity is due to the form of the Eqs. (11) and (43), which are very complicated expressions of $\textbf{k}$, involving thus, many processes that depends on $\textbf{k}$. For the Hermitian model on Lieb lattice, we must have the canceling of some terms in Eq. (4) however, the expression for $\sigma ^{reg}(\omega )$ does not change a lot and hence, the behavior for the conductivity at $\omega \rightarrow 0$ must be the same. Furthermore, as we obtain a finite Drude’s weight for all values of *T*, we have a Dirac’s delta peak for the conductivity at $\omega =0$ and consequently, we obtain that the transport is ideal in this point ($\omega =0$) for all values of *T*. For values nonzero of $\omega$ ($\omega \ne 0$), we obtain a decreasing in the conductivity for higher values of *T* and $\omega$, although this behavior is only qualitative due to approach used.

## Summary

In brief, we analyze the transport for the 2D non-Hermitian Lieb lattice and Ising model which are important models of quantum dissipative systems. The analysis for the XXZ model may be made in a future work. As far as I know, there is none experimental result that investigates the influence of energy bands on spin conductivity for the non-Hermitian models considered here. However, the rapid advance of experimental techniques in the last years has allowed the study of many systems in more complex lattices geometries^41, 51–57^. In a general way, in quantum spin systems, either real fields or complex fields generate a splitting of the degenerate ground states, where the spins are aligned along of the direction of the external magnetic field. The eigenvalues and the eigenvectors of the system with real spectrum do not change with the external magnetic field and in general, the initial state display a oscillating behavior and periodic among all possible spin configurations. This situation change a lot when a critical complex field is applied. The eigenstates and the dynamical behavior suffer a large change where all the initial states evolve to a coalescent state independent of the initial spin configurations. Thus, it is interesting to obtain the intriguing features of a quantum spin system in the presence of complex fields.

## Data Availability

All data generated or analysed during this study are included in this paper.
